# Ancestral Spectrum Analysis With Population-Specific Variants

**DOI:** 10.3389/fgene.2021.724638

**Published:** 2021-09-27

**Authors:** Gang Shi, Qingmin Kuang

**Affiliations:** State Key Laboratory of Integrated Services Networks, Xidian University, Xi’an, China

**Keywords:** admixture, population-specific SNP, rare variants, best linear unbiased estimator, ancestry inference

## Abstract

With the advance of sequencing technology, an increasing number of populations have been sequenced to study the histories of worldwide populations, including their divergence, admixtures, migration, and effective sizes. The variants detected in sequencing studies are largely rare and mostly population specific. Population-specific variants are often recent mutations and are informative for revealing substructures and admixtures in populations; however, computational methods and tools to analyze them are still lacking. In this work, we propose using reference populations and single nucleotide polymorphisms (SNPs) specific to the reference populations. Ancestral information, the best linear unbiased estimator (BLUE) of the ancestral proportion, is proposed, which can be used to infer ancestral proportions in recently admixed target populations and measure the extent to which reference populations serve as good proxies for the admixing sources. Based on the same panel of SNPs, the ancestral information is comparable across samples from different studies and is not affected by genetic outliers, related samples, or the sample sizes of the admixed target populations. In addition, ancestral spectrum is useful for detecting genetic outliers or exploring co-ancestry between study samples and the reference populations. The methods are implemented in a program, Ancestral Spectrum Analyzer (ASA), and are applied in analyzing high-coverage sequencing data from the 1000 Genomes Project and the Human Genome Diversity Project (HGDP). In the analyses of American populations from the 1000 Genomes Project, we demonstrate that recent admixtures can be dissected from ancient admixtures by comparing ancestral spectra with and without indigenous Americans being included in the reference populations.

## Introduction

With advances in sequencing technology, an increasing number of populations have been sequenced to study the histories of worldwide populations, including their divergence, admixtures, migration and effective sizes (Mallick et al., 2016; Pagani et al., 2016; Bergström et al., 2020). The variants detected in sequencing studies are largely rare and mostly population specific. In the 1000 Genomes Project (1kGP) dataset released in phase 3, 86% of the 88 million variants are restricted to a single continental population, and approximately 64 million autosomal variants have minor allele frequencies (MAFs) of less than 0.5% (Auton et al., 2015). Although population-specific variants are often recent mutations and are informative for revealing substructures and admixtures in populations (Auton et al., 2015), no methods have been developed for exploiting the enriched information in them.

Methods using rare variants for ancestry inference or analysis of population structures are also limited. Prokopenko et al. (2016) constructed a genetic similarity matrix based on the Jaccard similarity index. They demonstrated that eigenanalysis of the similarity matrix works particularly well for variants with small MAFs and provides a higher resolution of population substructure than that obtained with classical principal component analysis (PCA). Turkmen et al. (2019) considered four types of genetic similarity matrices that were analyzed by classical PCA, generalized PCA and logistic PCA. Ancestry memberships were predicted by a support vector machine using the top principal components (PCs), and logistic PCA was shown to have the highest classification accuracy for rare and low-frequency variants.

In this work, we propose using reference populations and single nucleotide polymorphisms (SNPs) specific to the reference populations. The eigenvalues and eigenvectors of the genetic relationship matrix based on population-specific SNPs (GRM-PS) are shown to be population specific in the reference populations. When analyzing the study samples, the principal scores associated with the reference populations are computed by projecting the genotype matrix onto the asymptotic principal directions defined by the reference populations. The principal scores are shown to be unbiased estimates of the ancestral proportions of maximum variances. Ancestral information, the best linear unbiased estimator (BLUE) of the ancestral proportion, is proposed, which can be used to infer ancestral proportions in recently admixed target populations and measure the extent to which reference populations serve as good proxies for the admixing sources. The methods are implemented in a program, Ancestral Spectrum Analyzer (ASA) available at https://github.com/eat1000/ASA, and are applied in analyzing high-coverage sequencing data from the 1kGP (Byrska-Bishop et al., 2021) and the Human Genome Diversity Project (HGDP)—Centre d’Etude du Polymorphisme Humain (CEPH) panel (Bergström et al., 2020). In the analyses of American populations from the 1kGP, we demonstrate that recent admixtures can be dissected from ancient admixtures by ancestral spectra with and without indigenous Americans being included in the reference populations.

## Materials and Methods

PCA-based methods have been developed for ancestry inference (Wang et al., 2015; Zhang et al., 2020) and the connection between eigenvectors and ancestral proportions was established (Ma and Amos, 2010). We will first consider the PCA with population-specific SNPs and show that the principal scores are unbiased estimates of the ancestral proportions with maximum variances. We will then propose a new score, which we term ancestral information, and demonstrate that it is the BLUE of the ancestral proportion with minimum variance. We will show that the computation of principal scores and ancestral information requires only the MAFs of population-specific SNPs in the reference populations, which does not involve the eigenanalysis or singular value decomposition as in the PCA-based methods (Wang et al., 2015; Zhang et al., 2020) or solving the likelihood model as in the model-based approaches, such as ADMIXTURE (Alexander et al., 2009; Alexander and Lange, 2011).

### Genetic Relationship Matrix Based on Population-Specific SNPs

Considering a sample of individuals from *K* reference populations, there are *N_k_* individuals from population *k*, *k* = 1,2,⋯,*K*, and the total sample size is *N* = *N*_1_ + *N*_2_ + ⋯ + *N*_*K*_. We assume that there is a panel of *M* biallelic SNPs that are population specific; that is, each SNP is polymorphic in one and only one of the *K* reference populations. Suppose that *𝔊*_*k*_ is the index set of SNPs that are polymorphic in population *k* and *M*_*k*_ = |*𝔊*_*k*_| is the number of SNPs specific to population *k*; then, the total SNP number is *M* = *M*_1_ + *M*_2_ + ⋯ + *M*_*K*_. Let *f*_*km*_ be the MAF of SNP *m* in population *k*; then, we have *f*_*km*_ > 0 if *m* ∈ *𝔊*_*k*_, and otherwise, *f*_*km*_ = 0.

Let ***X*** be a genotype matrix of dimension *N*×*M* whose elements ***X***(*n,m*) are coded as the number of minor alleles of SNP *m* in individual *n*. Suppose that *𝔖*_*k*_ is the index set of individuals who belong to population *k*, *k* = 1,2,⋯,*K*. For rare SNPs, ***X***(*n,m*) approximately follows a binomial distribution that takes values 0 and 1 with probabilities 1−2*f*_*km*_ and 2*f*_*km*_, respectively, provided that *n* ∈ *𝔖*_*k*_ and *m* ∈ *𝔊*_*k*_. It is easy to show that the genotypic mean and variance are


$$\mu_{k\,m}=\text{E}\left[\text{X}\left(n,m\right)|n\in {𝔖}_{k},m\in {𝔊}_{k}\right]=2f_{k\,m},$$

$$\sigma_{k\,m}^{2}=\text{Var}\left[\text{X}\left(n,m\right)|n\in {𝔖}_{k},m\in {𝔊}_{k}\right]=2f_{k\,m}\left(1-2f_{k\,m}\right).$$

For convenience, we assume that the rows of ***X*** are ordered by the populations to which the individuals belong, and the columns are ordered by the populations to which the SNPs are specific. That is, the first *N*_1_ rows are genotypic values of individuals from population 1, the next *N*_2_ rows are from population 2, and so on. Similarly, the first *M*_1_ columns are SNPs that are polymorphic in population 1, the next *M*_2_ columns are SNPs that are polymorphic in population 2, and so on. Since all SNPs are population specific, ***X***(*n,m*) is zero if *n* ∈ *𝔖*_*k*_, $m\in {𝔊}_{k^{\prime }}$ and *k*≠*k*′. Therefore, ***X*** is a block-diagonal matrix


$$\text{X}=\left(\begin{array}{cccc} {\text{X}}_{1} & 0 & \cdots & 0\\ 0 & {\text{X}}_{2} & \cdots & 0\\ \cdots & \cdots & \ddots & \cdots \\ 0 & 0 & \cdots & {\text{X}}_{K} \end{array}\right),$$

where ***X**_k_* is of dimensions *N_k_* = *M_k_*, *k* = 1,2,⋯,*K*.

We define the GRM-PS as


(1) $$\text{Z}=\frac{1}{M}\,{\text{XX}}^{\text{T}}.$$

Clearly, the GRM-PS ***Z*** is a block-diagonal matrix of dimension *N*×*N*,


(2) $$\text{Z}=\left(\begin{array}{cccc} {\text{Z}}_{1} & 0 & \cdots & 0\\ 0 & {\text{Z}}_{2} & \cdots & 0\\ \cdots & \cdots & \ddots & \cdots \\ 0 & 0 & \cdots & {\text{Z}}_{K} \end{array}\right),$$

where


$${\text{Z}}_{k}=\frac{1}{M}\,{\text{X}}_{k}\,{\text{X}}_{k}^{\text{T}},$$

$$Z{}_{k}\left(i^{\prime },i^{\prime }\right)=\frac{1}{M}\sum_{m=1}^{M}X{}^{2}\left(i,m\right)=\frac{1}{M}\sum_{m\in {𝔊}_{k}}X{}^{2}\left(i,m\right),i\in {𝔖}_{k},$$

$$Z{}_{k}\left(i^{\prime },j^{\prime }\right)=\frac{1}{M}\sum_{m=1}^{M}X\left(i,m\right)X\left(j,m\right)=\frac{1}{M}\sum_{m\in {𝔊}_{k}}X\left(i,m\right)X\left(j,m\right),i,j\in {𝔖}_{k},i\neq j.$$

*i* and *j* are the indices of individuals *i* and *j* in **Z**, respectively, and *i*′ and *j*′ are their indices in ***Z**_k_*.

Since the GRM-PS **Z** is block-diagonal, the eigenvalues of submatrix ***Z**_k_* are the eigenvalues of **Z**, and the associated eigenvectors of ***Z**_k_* padded with $\sum_{i=1}^{k-1}N_{i}$ and $\sum_{i=k+1}^{K}N_{i}$ zeros before and after, respectively, are the corresponding eigenvectors of **Z**. Therefore, the eigenvalues and eigenvectors of the GRM-PS are population specific in the reference populations.

### Expected Genetic Relationship Matrix Based on Population-Specific SNPs

According to the law of large numbers, as the SNP numbers *M*_1_,*M*_2_,⋯,*M*_*K*_ become large, the GRM-PS will converge to its mathematical expectation, that is, the expected GRM-PS (EGRM-PS). The EGRM-PS *Z* is also a block-diagonal matrix


(3) $$\text{Z}=\left(\begin{array}{cccc} {\text{Z}}_{1} & 0 & \cdots & 0\\ 0 & {\text{Z}}_{2} & \cdots & 0\\ \cdots & \cdots & \ddots & \cdots \\ 0 & 0 & \cdots & {\text{Z}}_{K} \end{array}\right),$$

where submatrices *Z_k_* is the expectation of ***Z**_k_*, *k* = 1,2,⋯,*K*.

Moreover, *Z_k_* is compound symmetric


$${\text{Z}}_{k}=\left(\begin{array}{cc} \begin{array}{cc} z^{k} & z^{k\,k}\\ z^{k\,k} & z^{k} \end{array} & \begin{array}{cc} \cdots & z^{k\,k}\\ \cdots & z^{k\,k} \end{array}\\ \begin{array}{cc} \vdots & \vdots \\ z^{k\,k} & z^{k\,k} \end{array} & \begin{array}{cc} \ddots & \vdots \\ \cdots & z^{k} \end{array} \end{array}\right),$$

where


(4) $$z^{k}=\text{E}\,\left[{\text{Z}}_{k}\,\left(i^{\prime },i^{\prime }\right)\right]=\frac{1}{M}\,\sum_{m\in {𝔊}_{k}}\text{E}\,\left[{\text{X}}^{2}\,\left(i,m\right)\right]=\frac{1}{M}\,\sum_{m\in {𝔊}_{k}}2\,f_{k\,m},$$

(5) $$z^{k\,k}=\text{E}\,\left[{\text{Z}}_{k}\,\left(i^{\prime },j^{\prime }\right)\right]=\frac{1}{M}\,\sum_{m\in {𝔊}_{k}}\text{E}\,\left[\text{X}\,\left(i,m\right)\,\text{X}\,\left(j,m\right)\right]=\frac{1}{M}\,\sum_{m\in {𝔊}_{k}}4\,f_{k\,m}^{2}.$$

### Eigenanalysis of the Expected GRM-PS

An eigenanalysis of the EGRM-PS *Z* provides the asymptotic results of the eigenanalysis of the GRM-PS **Z** when *M*_1_,*M*_2_,⋯,*M*_*K*_ are large. Submatrix *Z*_*kk*_ has the largest eigenvalue *λ*_*k*_ = *z^k^* + (*N*_*k*_−1)*z*^*kk*^ with the associated eigenvector $1_{N_{k}}/\sqrt{N_{k}}$ (Johnson and Wichern, 2007), where **1**_*N*_*k*__ is the column vector of dimension *N_k_* in which each element is 1. The eigenvalue *λ*_*k*_ can be decomposed as


$$\lambda_{k}=N_{k}\,z^{k\,k}+z^{k}-z^{k\,k}$$

(6) $$=\frac{N_{k}}{M}\,\sum_{m\in {𝔊}_{k}}\mu_{k\,m}^{2}+\frac{1}{M}\,\sum_{m\in {𝔊}_{k}}\sigma_{k\,m}^{2}.$$

The first component of *λ*_*k*_ depends on the MAFs of SNPs specific to population *k*, and the second consists of the intrapopulation variance of the SNPs. When plotting the PC associated with *λ*_*k*_, that is the *k*-th eigenvector scaled by $\sqrt{M\,\lambda_{k}}$ , the individuals from population *k* share the same coordinates, which represent the center of population *k* in this dimension (Ma and Amos, 2010). In real data analysis, the coordinates of the individuals from population *k* are distributed around the center and will converge to it as *M_k_* increases.

The other *N_k-1_* eigenvalues of *Z*_*kk*_ have the same value (Johnson and Wichern, 2007), which is


(7) $$z^{k}-z^{k\,k}=\frac{1}{M}\,\sum_{m\in {𝔊}_{k}}\sigma_{k\,m}^{2}.$$

Note that the eigenvectors associated with the repeated eigenvalue *z^k^*−*z*^*kk*^ are not unique and that the eigenvalue depends solely on the intrapopulation variance. Hence, their PCs have nothing to do with the genetic distances among individuals and contain no information about the population structure. In summary, among the *N* eigenvalues and eigenvectors of the EGRM-PS, *K* eigenvalues *λ*_1_,*λ*_2_,⋯,*λ*_*K*_ and their associated eigenvectors are population informative and population specific.

### Principal Score Vectors

In classical PCA, determining the top PCs involves eigenanalysis of the genetic relationship matrix (Patterson et al., 2006; Price et al., 2006) or singular value decomposition of the standardized genotype matrix (Galinsky et al., 2016). It can be shown that when *M_k_* and *N_k_* are large in the reference populations, the right singular vector of **X**, or the principal direction, associated with population *k* is


(8) $${\text{v}}_{k}={\left[\mu_{k\,1},\mu_{k\,2},\cdots ,\mu_{k\,M}\right]}^{\text{T}}/\sqrt{\sum_{m=1}^{M}\mu_{k\,m}^{2}}.$$

Assume that *f*_*km*_, *k* = 1,2⋯*K* and *m* = 1,2⋯*M*, are known or can be accurately estimated in the reference populations. Without eigenanalysis or singular value decomposition, the PC specific to population *k* can be computed by projecting the genotype matrix **X** onto the principal direction ***v**_k_*:


(9) $${\text{Xv}}_{k}=s_{k}\,{\text{u}}_{k},$$

and when *M_k_* is large,


(10) $$s_{k}=\sqrt{N_{k}\,\sum_{m=1}^{M}\mu_{k\,m}^{2}},$$

(11) $${\text{u}}_{k}={\left[0_{N_{1}}^{\text{T}},\cdots ,0_{N_{k-1}}^{\text{T}},1_{N_{k}}^{\text{T}},0_{N_{k+1}}^{\text{T}},\cdots ,0_{N_{K}}^{\text{T}}\right]}^{\text{T}}/\sqrt{N_{k}}.$$

Here, *s_k_* and ***u**_k_* are the singular value and left singular vector associated with population *k*. Note that ***u**_k_* is also the eigenvector of the EGRM-PS *Z* that is specific to population *k* and depends on the population size *N_k_*.

We define the principal score vector ***a**_k_* for population *k*, *k* = 1,2⋯*K*, as


(12) $${\text{a}}_{k}={\text{Xb}}_{k},$$

where


(13) $${\text{b}}_{k}={\left[\mu_{k\,1},\mu_{k\,2},\cdots ,\mu_{k\,M}\right]}^{\text{T}}/\sum_{m=1}^{M}\mu_{k\,m}^{2}.$$

It can be shown that ***a**_k_* will converge to ${\left[0_{N_{1}}^{\text{T}},\cdots ,0_{N_{k-1}}^{\text{T}},1_{N_{k}}^{\text{T}},0_{N_{k+1}}^{\text{T}},\cdots ,0_{N_{K}}^{\text{T}}\right]}^{\text{T}}$ in probability as *M_k_* becomes large. Therefore, individuals from population *k* are expected to have values of one, while individuals from other populations have values of exactly zero. Proofs are presented in the Supplementary Text.

For samples that include admixed individuals, the block structure of the GRM-PS does not hold, and the PCs are no longer population specific. However, the principal scores can still be computed. Admixed individuals carry alleles specific to multiple reference populations and will have more than one non-zero principal score. Therefore, the *K* principal score vectors provide a dissection of the population structure in the study samples. Since the loading vector ***b**_k_* is based on the asymptotic right singular vector ***v**_k_*, which aims to maximize the singular value *s_k_* in the reference populations, it weights SNPs with larger MAFs higher. In the Supplementary Text, we show that the principal scores are unbiased estimates of the ancestral proportions with maximum variances.

Note that ***b**_k_* depends on the MAFs of population-specific SNPs in the preselected reference populations, genotypes of one individual under analysis do not affect the principal score of another one. Samples from different studies can be inferred based on the same panel of SNPs, and the results are directly comparable. Moreover, since all individuals are compared in the asymptotic principal directions defined by the reference populations, genetic outliers, related samples and the sample sizes of the studies will not affect the results. On the other hand, for estimating MAFs of the population-specific SNPs, genetic outliers or related samples in the reference populations still need to be avoided and large sample sizes are desirable.

### Ancestral Information Vectors

Suppose that individual *n* is an admixed individual whose ancestral populations are in the *K* reference populations. Let $p_{n}^{1},p_{n}^{2},\cdots ,p_{n}^{K}$ denote the ancestral proportions of individual *n* for the *K* populations. Assume that individual *n* inherits two haplotypes at the locus of SNP *m* independently from the *K* populations. Considering one haplotype, it has probability $p_{n}^{k}$ originating from population *k*. Given that the haplotype is from population *k*, it has probability *f*_*km*_ carrying the allele specific to population *k*. Assuming statistical independence among the *M* SNPs in view of their low MAFs that lead to weak linkage disequilibrium with other rare or common variants (Prokopenko et al., 2016), the genotype of individual *n* follows the distribution


(14) $$p\,\left(\text{X}\,\left(n,1\right),\cdots ,\text{X}\,\left(n,m\right)\right)=\prod_{k=1}^{K}\prod_{m\in {𝔊}_{k}}{\left(2\,p_{n}^{k}\,f_{k\,m}\right)}^{X\,\left(n,m\right)}\,{\left(1-2\,p_{n}^{k}\,f_{k\,m}\right)}^{1-X\,\left(n,m\right)},$$

where **X**(*n*,*m*) = 0,1.

Without the constraint $\sum_{k=1}^{K}p_{n}^{k}=1$ , the estimate of $p_{n}^{k}$ obtained by the method of moment is


(15) $${\hat{p}}_{n}^{k}=\frac{\sum_{m\in {𝔊}_{k}}\text{X}\,\left(n,m\right)}{\sum_{m\in {𝔊}_{k}}\mu_{k\,m}},$$

where *k* = 1, 2,⋯,*K*. The numerator is the number of alleles specific to population *k* that are observed in individual *n*, and the denominator is the expected number of alleles for individuals from population *k*. The maximum likelihood estimate of $p_{n}^{k}$ can be obtained by solving the equation


(16) $$p_{n}^{k}\,\sum_{m\in {𝔊}_{k}}\mu_{k\,m}\,\frac{1-\text{X}\,\left(n,m\right)}{1-p_{n}^{k}\,\mu_{k\,m}}-\sum_{m\in {𝔊}_{k}}\text{X}\,\left(n,m\right)=0.$$

Proofs and an approximate maximum likelihood estimate are presented in the Supplementary Text.

We define the ancestral information vector for population *k* as follows:


(17) $${\text{c}}_{k}={\text{Xd}}_{k},$$

where


(18) $${\text{c}}_{k}={\left[{\hat{p}}_{1}^{k},{\hat{p}}_{2}^{k},\cdots \,{\hat{p}}_{N}^{k}\right]}^{\text{T}},$$

(19) $${\text{d}}_{k}={\left[0_{M_{1}}^{\text{T}},\cdots ,0_{M_{k-1}}^{\text{T}},1_{M_{k}}^{\text{T}},0_{M_{k+1}}^{\text{T}},\cdots ,0_{M_{K}}^{\text{T}}\right]}^{\text{T}}/\sum_{m=1}^{M}\mu_{k\,m}.$$

In the Supplementary Text, we show that the ancestral information is the BLUE of the ancestral proportion when MAFs of the SNPs are small. Compared with the loading vector ***b**_k_* for computing the principal score vector, ***d**_k_* weights SNPs equally. Similar to the principal score, ancestral information from different studies can be inferred based on the same panel of SNPs, and the results are comparable. Genetic outliers, related samples and the sample sizes of the target populations will not affect the results. It is worth emphasizing that interpreting the ancestral information as the ancestral proportion is valid only if its associated reference population approximates an ancestral population of the study sample well. Otherwise, it simply measures the co-ancestry between the reference population and the study sample.

### The 1000 Genomes Project Dataset

The samples and the high-coverage sequencing data of the 1kGP were described previously (Auton et al., 2015; Byrska-Bishop et al., 2021). We extracted SNPs from the genotype data in VCF format and removed SNPs with multicharacter allele using PLINK 1.9 (Chang et al., 2015). Autosomal SNPs having a reference SNP number in dbSNP build 151 (Sherry et al., 2001) were retained, which included 95,250,105 biallelic SNPs. The 2,504 unrelated individuals of the 1kGP are from African (AFR), American (AMR), East Asian (EAS), European (EUR), and South Asian (SAS) populations. We excluded individuals from recently admixed populations, which are AMRs known to have European, African and indigenous American ancestries as well as AAs of primarily African and European ancestries (Auton et al., 2015). We selected EUR, EAS, SAS, and AFR populations as the reference populations, each with a sample size of approximately 500. SNPs that were polymorphic in exactly one of the four reference populations were identified, and the numbers of population-specific SNPs in different MAF ranges are shown in Table 1. We then randomly selected 50,000 SNPs with MAFs between 0.01 and 0.05 in the respective populations, which made up a panel of 200,000 SNPs specific to AFR, EAS, EUR, or SAS. The SNPs and their MAFs in the reference populations are presented in Supplementary Table 1.

**TABLE 1:** Summary of population-specific SNPs in the 1kGP dataset.

Population	N	Population specific	Singleton	MAF > 0.5%	MAF > 1%	MAF > 5%	MAF > 10%
AFR	504	24,676,870	10,948,012	7,480,488	5,322,732	1,329,784	410,704
EAS	504	13,699,338	9,303,301	1,077,695	475,273	17,801	1,162
EUR	503	10,184,006	7,679,086	500,612	155,604	540	0
SAS	489	12,213,864	7,929,391	1,404,054	571,614	18,651	1,004
Total	2000	60,774,078	35,859,790	10,462,849	6,525,223	1,366,776	412,870

### The Human Genome Diversity Project Dataset

The HGDP-CEPH panel consists of 929 high-coverage human genome sequences from 54 diverse populations throughout the world (Bergström et al., 2020). The 929 individuals originate from seven geographic regions: Africa, America, Central and South Asia, East Asia, Europe, the Middle East, and Oceania. The SNPs were extracted by PLINK 1.9, and those with multicharacter alleles were removed. Autosomal SNPs with reference SNP numbers were retained, which yielded 55,560,915 biallelic SNPs. Combining the indigenous AMR populations (*N* = 61) from the HGDP-CEPH with AFR, EAS, EUR, SAS populations from the 1kGP, we obtained a set of five reference populations. There were 161,088 SNPs specific to AMR, and the numbers of SNPs specific to AFR, EAS, EUR, and SAS were slightly smaller than those in Table 1. A second panel of population-specific SNPs of size 250,000 was constructed with 50,000 SNPs specific to each of the five reference populations. For SNPs specific to AFR, EAS, EUR, and SAS, their MAFs were between 0.01 and 0.05 in the respective populations. SNPs specific to AMR were chosen with MAFs between 0.025 and 0.15 considering the small sample size of the AMR populations and the small number of SNPs specific to AMR. The panel of SNPs and their MAFs in the reference populations are presented in Supplementary Table 2.

## Results

### Principal Components and Principal Scores in the 1kGP

Using the 200,000 populations-specific SNPs, we conducted PCA on the reference populations, which included 2,000 non-admixed individuals. Scatter plots of the top four PCs are shown in Supplementary Figure 1. We can see that PC 1 is specific to AFR, and all individuals from EAS, EUR, and SAS have values of zero. Similarly, PCs 2, 3, and 4 are specific to EAS, SAS, and EUR, respectively. Because each of the four reference population groups consists of five populations, additional PCs associated with smaller eigenvalues are informative for population substructures. PCs 5, 6, 7, and 9 were found to be specific to EAS, EUR, AFR, and SAS, respectively, as shown in Supplementary Figure 2. Including admixed individuals in the PCA, the PCs are no longer population specific. Supplementary Figure 3 presents the top four PCs of 2,504 individuals, which includes individuals from the AMR and African American (AA) populations. Compared with Supplementary Figure 1, the PCs are rotated due to the correlations introduced by the admixed individuals.

**FIGURE 1:**
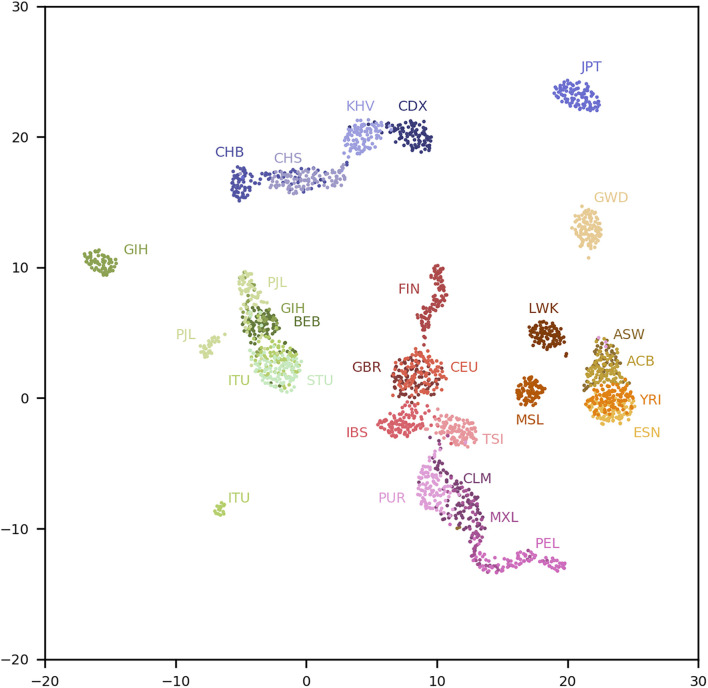
UMAP analysis of the top 20 PCs in the 1kGP using four reference populations. Population labels are explained in the Supplementary Text.

We conducted a uniform manifold approximation and projection (UMAP) 0.4.3 analysis (McInnes et al., 2018) on the top 20 PCs using a minimum distance of 1 and 100 nearest neighbors. The results are shown in Figure 1. Fine-scale population structures are revealed by the population-specific SNPs. Although the MAFs of the 200,000 population-specific SNPs are low and the top 20 PCs explain only 6.3% of the genotypic variance, the resolution of the population structure is comparable with that obtained by using SNPs from Affymetrix 6.0 (Diaz-Papkovich et al., 2019). For comparison, we randomly selected 200,000 common SNPs (MAFs > 0.1) in the total population and conducted a classical PCA, whose top 20 PCs explained 17.2% of the genotypic variance. A UMAP analysis with the same parameters is shown in Supplementary Figure 4. As shown, the populations are better separated with population-specific SNPs. For example, populations CDX and KHV form separated clusters except for a few individuals in Figure 1, while they moderately overlap in Supplementary Figure 4. Similar observations can be made for the IBS and TSI populations.

The principal scores of the 2,504 individuals are shown in Supplementary Figure 5. Individuals from the reference populations have one non-zero score that is associated with the population to which they belong. AAs and AMRs have multiple non-zero scores associated with the reference populations. Since the loading vectors of the principal scores depend on the MAFs of the population-specific SNPs only, including admixed individuals does not change the scores of individuals from the reference populations.

### Ancestral Spectra of the 1kGP Using Four Reference Populations

**FIGURE 2:**
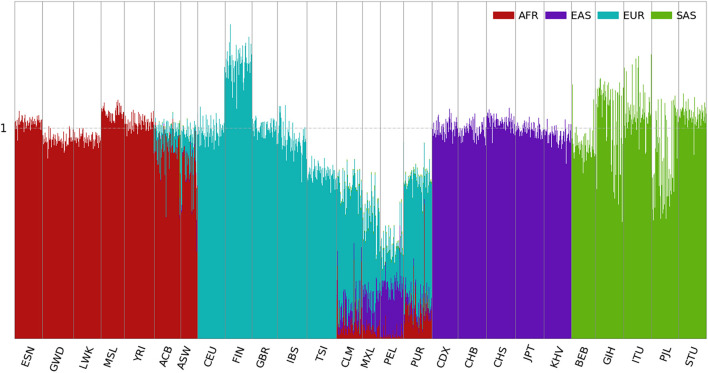
Ancestral spectra of 2,504 individuals in the 1kGP using four reference populations. Population labels are explained in the Supplementary Text.

The ancestral spectra of the 2,504 individuals are shown in Figure 2. Since we chose AFR, EAS, EUR, and SAS in the 1kGP as the reference populations, unsurprisingly, individuals from the four populations showed a single ancestral component. The scatter plots of the ancestry information are presented in Supplementary Figure 6, and the individual results are shown in Supplementary Table 3. The ancestral information in Supplementary Figure 6 is close to the principal scores in Supplementary Figure 5, because they are all unbiased estimates of the ancestral proportions. Although individuals from the same reference population are expected to have ancestral information of value one for this population, variation can be observed across populations. For instance, individuals from FIN have an average EUR information of 1.31, while it is 0.79 in TSI. This is due to MAF differences in different EUR populations. The average MAFs of the 50,000 EUR-specific SNPs in FIN and TSI are 0.021 and 0.013, respectively, compared with 0.016 in EUR.

For the admixed populations, summary statistics of their AFR, EAS, EUR, and SAS information are presented in Supplementary Figure 7. The two AA populations, ACB and ASW, have average AFR information of 0.895 and 0.776 and average EUR information of 0.097 and 0.167, respectively. In addition, a small number of EAS components are present in ASW, likely because of the admixture with indigenous AMRs. The estimated AFR and EUR information in ACB and ASW are close to their admixture proportions reported in the literature (Bryc et al., 2010), and the sums of the two components are close to one. Since AAs are mostly recent admixtures of African and European ancestries, it appears that contemporary AFR and EUR populations in the 1kGP serve as reasonable proxies of their ancestral populations.

For AMR populations, the average AFR, EAS, and EUR information varies in different populations. PUR shows the highest AFR (0.136), the lowest EAS (0.045) and the highest EUR information (0.577), and PEL shows the lowest AFR (0.033), highest EAS (0.232) and lowest EUR information (0.194). Because of the lack of an indigenous AMR population in the reference populations in this analysis, EAS serves as a proxy. Indigenous AMRs are considered to have migrated from Siberia via Beringia approximately 15,000–23,000 years ago (Nielsen et al., 2017). Because of the migration bottleneck, only 5,454 the 50,000 EAS-specific SNPs are polymorphic in the AMR populations. Therefore, the EAS information underestimates the admixture proportion for indigenous AMRs. On the other hand, the average EAS and EUR information in the indigenous AMR populations from the HGDP is 0.289 and 0.065, respectively, as shown in the latter part of the results. The EUR information of the AMR populations from the 1kGP includes some ancient admixtures before the Columbian contact.

Some genetic outliers can be observed whose ancestral spectra deviate from the distribution centers of the populations with which they are labeled. For example, NA20314 from ASW has AFR, EAS, and EUR information of 0.007, 0.203, and 0.316, respectively, and has almost no AFR ancestry. HG01880 from ACB has AFR, EAS, EUR, and SAS information of 0.570, 0.018, 0.079, and 0.369, respectively, and is the only member of AA showing substantial SAS ancestry. HG01241 from PUR has AFR, EAS, and EUR information of 0.693, 0.024, and 0.212, respectively, and is closer to AAs. It is interesting to note that the total ancestral information of NA20314 is only 0.531, which increases to 0.934 when the second SNP panel was used, see latter part of the results. This is because that she has a lot of AMR ancestry which was not well-represented by the EAS-specific SNPs in the first panel. With the second SNP panel, NA20314 has AFR, AMR, EAS, and EUR information 0.007, 0.588, 0.017, and 0.321, respectively.

### Ancestral Spectra of the 1kGP Using Five Reference Populations

**FIGURE 3:**
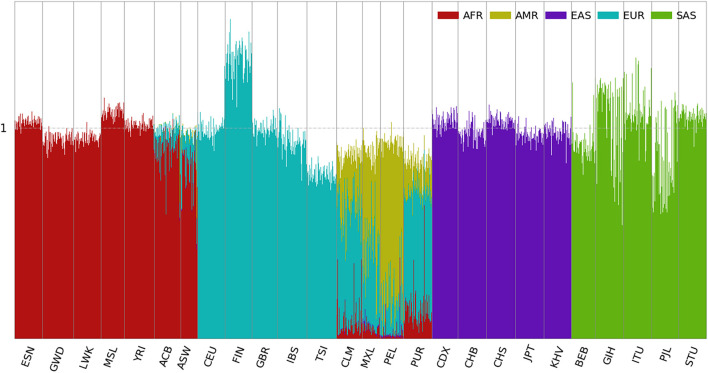
Ancestral spectra of 2,504 individuals in the 1kGP using five reference populations. Population labels are explained in the Supplementary Text.

**FIGURE 4:**
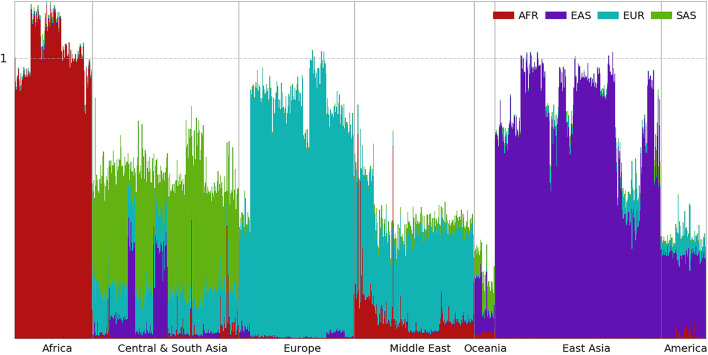
Ancestral spectra of 929 individuals in the HGDP using four reference populations.

**FIGURE 5:**
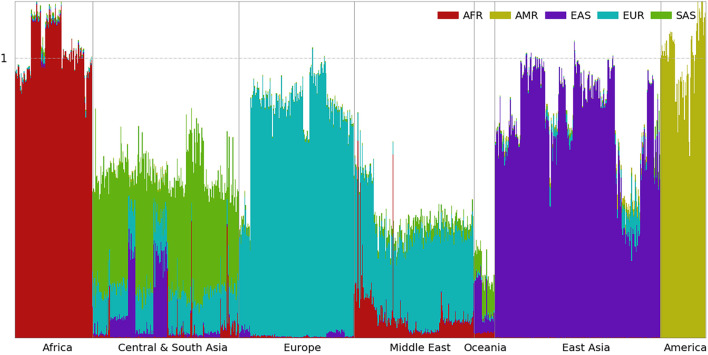
Ancestral spectra of 929 individuals in the HGDP using five reference populations.

Using the panel of 250,000 population-specific SNPs, ancestral spectra of the 2,504 individuals are shown in Figure 3, and individual results are shown in Supplementary Table 4. The average AMR information in CLM, MXL, PEL, and PUR is 0.257, 0.467, 0.711, and 0.139, respectively. EAS and EUR information decreased in the four AMR populations. For example, the average EAS and EUR information in CLM is 0.083 and 0.544 with the first panel of SNPs, which is reduced to 0.004 and 0.521, respectively, with the second panel. The EAS and EUR information that involves ancient admixtures in indigenous AMRs are now included in the AMR information, and the EUR information with the second SNP panel corresponds to recent admixtures only. The average EUR information due to the recent admixture after the Columbian contact is 0.418. Compared with 0.440 using the first SNP panel, the difference, about 0.022 EUR information, is attributable to their indigenous AMR ancestors. The EUR ancestry in the AMR ancestors might result from recent European admixture in the HGDP indigenous American groups (Hellenthal et al., 2014) and/or ancient admixture that happened in the ancestors of indigenous AMRs before their migration to America. The latter is supported by the presence of a similar degree of EUR ancestral information in HGDP populations from Siberia (Figures 4, 5). Summary statistics of the five ancestral information are presented in Supplementary Figure 8.

It is also interesting to note that for CLM and PUR, the EAS information is close to zero when analyzed with the second SNP panel. In contrast, some individuals from MXL and PEL still have a substantial amount of EAS ancestry. For example, HG01944 from PEL has AMR, EAS, and EUR information of 0.474, 0.377, and 0.101, respectively. The 0.101 EUR information should come from admixtures sometime after the Columbian contact and the 0.377 EAS information is possibly to be more recent. With the first SNP panel, the EAS information of HG01944 is 0.528. Because the 0.377 EAS information is from his contemporary EAS ancestor, the rest 0.151 is attributable to his indigenous AMR ancestor.

### Ancestral Spectra of the HGDP Using Four Reference Populations

In the first panel of 200,000 population-specific SNPs, 183,328 SNPs had at least one copy of rare alleles in the HGDP dataset. The ancestral information of the 929 individuals based on the first panel of SNPs is presented in Figure 4, and individual results are shown in Supplementary Table 5. European populations have the largest EUR information, which decreases in Central and South Asians and further decreases in East Asians. In contrast, the EAS information increases from west to east. Heterogeneous ancestral spectra can be observed in the Central and South Asian populations. The southernmost Sindhi population has the largest SAS information, 0.565, and the northernmost Uygur population has the least SAS information, 0.104. From east to west, the easternmost Uygur population has the largest EAS information, 0.349, and the westernmost Makrani population has the least EAS information, 0.006.

Oceanian populations have the least total ancestral information (0.251) from AFR, EAS, EUR, and SAS combined. This reflects their ancient separation and isolation from Eurasian populations (Nielsen et al., 2017). Due to the migration bottleneck, a small portion of rare alleles from the Eurasian populations were carried by their ancestors, which decreased further by genetic drift thereafter. Most of their ancestral information is from EAS (0.126) and SAS (0.098), and the least is from EUR (0.007), which is consistent with migration after the divergence between EUR and EAS (Nielsen et al., 2017). A small amount of the AFR component (0.020) can be observed in Oceanians. At the time of migration, some alleles specific to contemporary AFR populations might still be present in populations peopling South and East Asia.

The ancestral information of indigenous AMRs is mainly from EAS (0.289) and EUR (0.065). If ancestors of indigenous AMRs were admixed between EAS and EUR populations sometime before the migration of indigenous American ancestors across the Bering Strait, obviously, contemporary EAS the EUR populations are not good proxies of their ancestral populations and the ancestral information cannot be interpreted as the ancestral proportion. In fact, the chance of having good proxies using contemporary populations decreases as the admixture is more ancient. It is worth noting again that some of the EUR ancestry is likely due to recent admixture (Hellenthal et al., 2014). There is also a small amount of AFR ancestry in AMR, which mostly exists in the Mayan and Colombian populations and is almost absent in the Karitiana, Pima, and Surui populations, as reported previously (Hellenthal et al., 2014). HGDP00863 from the Mayan and HGDP00703 from the Colombian populations have AFR information of 0.047 and 0.053, respectively. This may be a result of admixture with AFR after the transatlantic slave trade (Micheletti et al., 2020). Summary statistics of the four ancestral information are shown in Supplementary Figure 9.

Inspecting the ancestral spectra of individuals, some genetic outliers can be identified. For example, HGDP00544 from the Papuan Sepik population has AFR, EAS, and SAS information values of 0.019, 0.217, and 0.089, respectively, which are closer to the average values of 0.014, 0.208, and 0.087 in the Bougainville population than the 0.023, 0.082, and 0.111 in the Papuan Sepik population. HGDP00871 from the Mayan population has EUR information of 0.245, which is much larger than the average of 0.065 in AMR. Similar to the AMR populations in the 1kGP, some EUR components of HGDP00871 may be due to recent admixture.

### Ancestral Spectra of the HGDP Using Five Reference Populations

Ancestral spectra with the second panel of 250,000 population-specific SNPs are shown in Figure 5, and individual results are shown in Supplementary Table 6. Since the AMR populations serve as the reference population for choosing AMR-specific SNPs, their ancestral components are fully attributed to AMR. Outside of America, the population showing the largest AMR information is Yakut (0.024) from eastern Siberia, close to the region from which the ancestors of indigenous AMR migrated. Some populations in East Asia that have small amounts of AMR information, which are Oroqen (0.020), Hezhen (0.014), Daur (0.013), and Mongolian (0.010) populations, likely due to the shared ancestors between northeastern Asian populations and AMR populations before divergence (Li et al., 2008). It may also due to gene flows between populations in America and eastern Siberia after the first migration of indigenous AMRs. The EAS information decreases slightly in these populations compared with the results using the first panel of SNPs. The AMR information for other populations is approximately zero. In Supplementary Figure 11, we also examine AFR, EAS, EUR, and SAS information with the two panels of population-specific SNPs in 868 non-American individuals. The four ancestral information computed by the two panels of SNPs are approximately the same.

## Discussion

Our ancestral information was derived based on the estimate of ancestral proportions. It can be interpreted as an ancestral proportion only if its associated reference population is recently related to an ancestral population, thus approximates the ancestral population well. For the recently admixed AAs in the 1kGP, contemporary AFR and EUR populations appear to serve as good proxies of their ancestral populations, and the results are close to the estimated ancestral proportions reported in literature (Bryc et al., 2010). However, for many other populations in the HGDP, the ancestral information simply measures the relative amount of rare alleles shared between the population under analysis and the respective reference population. Appropriate consideration of the population history has to be taken when interpreting mixed ancestries. The AMR component in the Yakut population can be explained by the shared ancestry between the Yakut population and the indigenous AMR populations or the gene flows between populations in America and eastern Siberia after the migration. For Oceanians, the mixed ancestries from EAS, SAS, and AFR suggest that the separation of Oceanians is ancient.

Technical issues may confound the estimation of ancestral information and prevent the comparison of absolute values between samples with different genotyping methods, such as sequencing platforms, sequencing coverage, reference genomes, quality control criteria, and bioinformatics software. Out of the first panel of 200,000 SNPs, we extracted 193,902 SNPs from the genotype data of the 1kGP released in phase 3. Ancestral spectrum analysis was conducted based on the 193,902 SNPs, and the results are shown in Supplementary Figure 12. The general patterns in Supplementary Figure 12 and Figure 2 are very similar, whereas the average total ancestral information of individuals with phase 3 data is 0.930, compared with 0.950 with high-coverage data and the same 193,902 SNPs. For the FIN population, the average EUR information was 1.256 with phase 3 data and 1.308 with high-coverage sequencing data. This reflects the greater sensitivity of high-coverage sequencing technology for calling rare alleles (Byrska-Bishop et al., 2021). Comparing the genotypes of 48,591 EUR-specific SNPs in the FIN population, an average of 1,945 and 2,031 EUR-specific alleles were carried per individual in the phase 3 and high-coverage data, respectively, and 94.92% of rare alleles in the high-coverage data were called in the phase 3 data. For the FIN population, there were also very small amounts of AFR, EAS, and SAS information with the phase 3 data. Among the 99 individuals, there were 144, 228, and 150 alleles specific to AFR, EAS, and SAS, respectively. Because the specificity of high-coverage sequencing is larger, most of these alleles are likely false discoveries. Detailed comparisons between the data from phase 3 and high-coverage sequencing can be found elsewhere (Byrska-Bishop et al., 2021).

Our selection of population-specific SNPs may be subject to misclassification errors. Alleles classified as specific to one population may have undetected and much lower frequencies in another population due to the limited sample sizes of the reference populations. For example, the average EAS, EUR, and SAS information in the Biaka population from the HGDP are 0.005, 0.007, and 0.008, respectively, which are unexpected. Increasing the sample sizes of the reference populations will reduce such errors. Since the ancestral information due to misclassification errors is typically very small, the overall ancestral spectrum will not be greatly affected. In addition, such a small amount of unexpected ancestral information can also be caused by sequencing errors.

We used AFR, EAS, EUR, and SAS populations from the 1kGP and AMR populations from the HGDP as the reference populations, some of which may be admixed as well. We conducted an unsupervised ADMIXTURE 1.3.0 (Alexander et al., 2009; Alexander and Lange, 2011) analysis on the 200,000 common SNPs with *K* = 4, 5, and the results are shown in Supplementary Figures 13, 14, respectively. The clusters were labeled with the continental populations that had the largest components. We can see that PJL shows not only SAS ancestry, but also EAS and EUR ancestries. Since PJL is one of the reference populations for SAS, rare alleles, if carried by PJL individuals and originating from EUR, were excluded from the panel of SNPs specific to EUR. As a result, the EUR ancestry of PJL, if present, was not detected and the number of SNPs specific to EUR was underestimated. Nevertheless, this is not a problem for analyzing populations outside of the reference populations. Due to the abundance of population-specific SNPs, genomic regions that carry alleles originating from EUR or EAS are covered by many other SNPs specific to the two populations. Therefore, our method is robust to the admixture in reference populations.

We further analyzed the 20 populations from the 1kGP that serve as reference populations for AFR, EAS, EUR, and SAS, the results are shown in Supplementary Figures 15, 16. In the analysis of one population, for instance ESN, we excluded it from the reference populations and used samples from GWD, LWK, MSL, and YRI as the reference population for AFR, while reference populations for the other population groups remained. Similar methods were used in the analyses of the other populations. Because ancestral spectra of the 20 populations were based on different panels of reference populations, absolute values of the ancestral information are comparable only within the same population. As we can see that despite mixed ancestries can be observed in the reference populations, populations from the same continental group share great majority of their ancestries.

Model-based approaches, such as STRUCTURE (Pritchard et al., 2000; Falush et al., 2003; Hubisz et al., 2009), FRAPPE (Tang et al., 2005), ADMIXTURE (Alexander et al., 2009; Alexander and Lange, 2011), and fastSTRUCTURE (Raj et al., 2014), have been widely used for analyzing population structure and inferring the ancestral proportions of individuals. For a given number of ancestral populations, allele frequencies in ancestral populations and the ancestral proportions of individuals are estimated simultaneously. Our ancestral information, on the other hand, is based on the allele frequencies of a panel of population-specific SNPs that are estimated *a priori*. The ancestral spectrum analysis in the study samples does not affect the estimates of allele frequencies in the reference populations; hence, genetic outliers and related individuals can be included in the target populations. Ancestral information from different studies can be inferred and compared based on the same panel of SNPs, and the results do not depend on the sample sizes of the studies.

Another difference between our ancestral information and the model-based approaches is that we do not constrain the total ancestral information to be one. This allows for comparing results with different reference populations. As illustrated in the analyses of AMR populations from the 1kGP, choosing different reference populations allows recent admixtures to be dissected from ancient ones. Moreover, our ancestral information is directly associated with one of the reference populations, and its interpretation is straightforward. Although we assumed that population-specific alleles are rare, it can be shown that the ancestral information estimated by the method of moment holds for low-frequency or common alleles as well. For the maximum likelihood estimate, the binomial approximation of the genotypic distribution is not valid any longer and the inference based on Eq. 14 will be less accurate. In addition, the estimator by the method of moment is the linear estimator that minimizes variance, is statistically consistent in nature and incurs minimal computational cost.

We conducted supervised ADMIXTURE 1.3.0 (Alexander et al., 2009; Alexander and Lange, 2011) analyses with the two sets of reference populations and population-specific SNPs used in the ancestral spectrum analyses, the results are shown in Supplementary Figures 17, 18. As can be seen in Supplementary Figure 17 that AMR groups have little EAS ancestry when the four reference populations were used. Presumably this is because the AFR and EUR proxies are much better for the true European and African source, respectively, than EAS is for the true indigenous American source. A smaller number of EAS-specific SNPs are polymorphic in AMRs than those of AFR or EUR-specific SNPs. All ancestry components add up to one for each individual because of the constraint on the total ancestral proportions. However, such constraint biases the estimates of AFR and EUR proportions upwardly. In Supplementary Figure 17, average AFR and EUR proportions are 0.082 and 0.914 in AMRs, respectively. Including indigenous AMR in the reference populations, the AFR and EUR proportions in Supplementary Figure 18 decrease to 0.070 and 0.485, respectively. Because of the constraint, the two sets of results cannot be compared meaningfully. The constraint may not be a problem when analyzing samples whose ancestral populations are known and exist in the reference populations (Lawson et al., 2018). For exploratory analyses that involve understudied populations, our unconstrained ancestral information provides an insight whether some ancestral sources are missing or poorly represented.

In this work, we use only five reference populations, which have publicly available deep sequencing data and reasonable sample sizes. With more populations sequenced in the future, more reference populations of larger sizes will be able to be used, and much finer ancestral spectra in worldwide populations will be able to be inferred.

## Data Availability Statement

Genotype data from high coverage sequencing data of the 1kGP are available from ftp://ftp.1000genomes.ebi.ac.uk/vol1/ftp/data_collections/1000G_2504_high_coverage. Genotype data from high coverage sequencing data of the HGDP are available from ftp://ngs.sanger.ac.uk/production/hgdp/hgdp_wgs.20190516/.

## Ethics Statement

Ethical review and approval was not required for the study on human participants in accordance with the local legislation and institutional requirements.

## Author Contributions

GS and QK: conceptualization, writing, review, and editing. GS: methodology, funding acquisition, supervision, and writing original draft. QK: visualization. Both authors contributed to the article and approved the submitted version.

## Conflict of Interest

The authors declare that the research was conducted in the absence of any commercial or financial relationships that could be construed as a potential conflict of interest.

## Publisher’s Note

All claims expressed in this article are solely those of the authors and do not necessarily represent those of their affiliated organizations, or those of the publisher, the editors and the reviewers. Any product that may be evaluated in this article, or claim that may be made by its manufacturer, is not guaranteed or endorsed by the publisher.
