# Supplementary material for: Ancestral Spectrum Analysis With Population-Specific Variants
Source: Front Genet. 2021 Sep 27;12:724638. doi: 10.3389/fgene.2021.724638 (PMC8503515; doi:10.3389/fgene.2021.724638)
Supplement: Supplementary file 1 [file Data_Sheet_1.PDF]

Ancestral Spectrum Analysis with Population-Specific Variants
— Supplementary Materials

Gang Shi[1*] and Qingmin Kuang[1]

[1]State Key Laboratory of Integrated Services Networks, Xidian University, 2 South Taibai Road, Xi'an, Shaanxi, 710071, China, GS: gshi@xidian.edu.cn, QK: qmkuang@stu.xidian.edu.cn

[*]Corresponding author

Supplementary Text

Population labels in the 1kGP

ACB, African Caribbean in Barbados

ASW, African Ancestry in Southwest US

BEB, Bengali in Bangladesh

CDX, Chinese Dai in Xishuangbanna, China

CEU, Utah residents with Northern and Western European ancestry

CHB, Han Chinese in Bejing, China

CHS, Southern Han Chinese, China

CLM, Colombian in Medellin, Colombia

ESN, Esan in Nigeria

FIN, Finnish in Finland

GBR, British in England and Scotland

GIH, Gujarati Indian in Houston, TX

GWD, Gambian in Western Division, The Gambia

IBS, Iberian populations in Spain

ITU, Indian Telugu in the UK

JPT, Japanese in Tokyo, Japan

KHV, Kinh in Ho Chi Minh City, Vietnam

LWK, Luhya in Webuye, Kenya

MSL, Mende in Sierra Leone

MXL, Mexican Ancestry in Los Angeles, California

PEL, Peruvian in Lima, Peru

PJL, Punjabi in Lahore,Pakistan

PUR, Puerto Rican in Puerto Rico

STU, Sri Lankan Tamil in the UK

TSI, Toscani in Italy

YRI, Yoruba in Ibadan, Nigeria

Proofs of equations (8)-(13)

To find the right singular vector of $\boldsymbol{X}$, or the principal direction, associated with population k, we maximize s_k^2 with respective to $\boldsymbol{v}_k$, where

$$s_k^2 = \|\boldsymbol{X}\boldsymbol{v}_k\|^2 = \sum_{n\in\mathfrak{S}_k}\left[\sum_{m\in\mathfrak{G}_k}\boldsymbol{X}(n,m)v_{km}\right]^2 \Big/ \sum_{m\in\mathfrak{G}_k} v_{km}^2.$$

Let

$$\frac{\partial s_k^2}{\partial v_{ki}} = 0,\ i \in \mathfrak{G}_k,$$

we have,

$$\sum_{n\in\mathfrak{S}_k} 2\left[\sum_{m\in\mathfrak{G}_k}\boldsymbol{X}(n,m)v_{km}\right]\boldsymbol{X}(n,i) \Big/ \sum_{m\in\mathfrak{G}_k} v_{km}^2 -$$

$$\sum_{n\in\mathfrak{S}_k}\left[\sum_{m\in\mathfrak{G}_k}\boldsymbol{X}(n,m)v_{km}\right]^2 2v_{ki} \Big/ \left[\sum_{m\in\mathfrak{G}_k} v_{km}^2\right]^2 = 0,$$

$$\left[\sum_{m\in\mathfrak{G}_k} v_{km}^2\right]\left\{\sum_{n\in\mathfrak{S}_k}\left[\sum_{m\in\mathfrak{G}_k}\boldsymbol{X}(n,m)v_{km}\right]\boldsymbol{X}(n,i)\right\} = \sum_{n\in\mathfrak{S}_k}\left[\sum_{m\in\mathfrak{G}_k}\boldsymbol{X}(n,m)v_{km}\right]^2 v_{ki}\,.$$

As M_k becomes large, $\frac{1}{M_k}\sum_{m\in\mathfrak{G}_k}\boldsymbol{X}(n,m)v_{km}$ will converge to its mathematical expectation $\frac{1}{M_k}\sum_{m\in\mathfrak{G}_k}\mu_{km}v_{km}$ according to the law of large numbers. Therefore, when M_k is large

$$\left[\sum_{m\in\mathfrak{G}_k} v_{km}^2\right]\left\{\sum_{n\in\mathfrak{S}_k}\left[\frac{1}{M_k}\sum_{m\in\mathfrak{G}_k}\mu_{km}v_{km}\right]\boldsymbol{X}(n,i)\right\} =$$

$$M_k\sum_{n\in\mathfrak{S}_k}\left[\frac{1}{M_k}\sum_{m\in\mathfrak{G}_k}\mu_{km}v_{km}\right]^2 v_{ki},$$

$\left[\sum_{m\in\mathfrak{G}_k} v_{km}^2\right]\left[\frac{1}{M_k}\sum_{m\in\mathfrak{G}_k}\mu_{km}v_{km}\right]\left[\sum_{n\in\mathfrak{S}_k}\boldsymbol{X}(n,i)\right] = M_k\left[\frac{1}{M_k}\sum_{m\in\mathfrak{G}_k}\mu_{km}v_{km}\right]^2 N_k v_{ki}$,

$\left[\sum_{m\in\mathfrak{G}_k} v_{km}^2\right]\left[\frac{1}{N_k}\sum_{n\in\mathfrak{S}_k}\boldsymbol{X}(n,i)\right] = v_{ki}\sum_{m\in\mathfrak{G}_k}\mu_{km}v_{km}$.

As N_k becomes large, $\frac{1}{N_k}\sum_{n\in\mathfrak{S}_k}\boldsymbol{X}(n,i)$ will converge to its mathematical expectation $\frac{1}{N_k}\sum_{n\in\mathfrak{S}_k}\mu_{ki} = \mu_{ki}$, hence, when N_k is large

$$\left[\sum_{m\in\mathfrak{G}_k} v_{km}^2\right]\mu_{ki} = v_{ki}\sum_{m\in\mathfrak{G}_k}\mu_{km}v_{km}. \qquad \text{(S-1)}$$

For $\frac{\partial s_k^2}{\partial v_{kj}} = 0$, $j \in \mathfrak{G}_k$ and $i \neq j$, we also have

$$\left[\sum_{m\in\mathfrak{G}_k} v_{km}^2\right]\mu_{kj} = v_{kj}\sum_{m\in\mathfrak{G}_k}\mu_{km}v_{km}. \qquad \text{(S-2)}$$

From (S-1) and (S-2), we have

$$\frac{\mu_{ki}}{\mu_{kj}} = \frac{v_{ki}}{v_{kj}}.$$

Therefore,

$$\boldsymbol{v}_k = [\mu_{k1}, \mu_{k2}, \cdots, \mu_{kM}]^{\mathrm{T}} / \sqrt{\sum_{m=1}^{M}\mu_{km}^2} \qquad \text{(S-3)}$$

is the *k*-th right singular vector of $\boldsymbol{X}$ when M_k and N_k are large.

The *k*-th PC in reference populations can be found by projecting genotype matrix $\boldsymbol{X}$ onto $\boldsymbol{v}_k$

$$\boldsymbol{X}\boldsymbol{v}_k$$

$$= \left[\sum_{m=1}^{M}\boldsymbol{X}(1,m)\mu_{km}, \sum_{m=1}^{M}\boldsymbol{X}(2,m)\mu_{km}, \cdots, \sum_{m=1}^{M}\boldsymbol{X}(N,m)\mu_{km}\right]^{\mathrm{T}} / \sqrt{\sum_{m=1}^{M}\mu_{km}^2}$$

$$= \left[\mathbf{0}_{N_1}^{\mathrm{T}}, \cdots, \mathbf{0}_{N_{k-1}}^{\mathrm{T}}, \sum_{m\in\mathfrak{G}_k}\boldsymbol{X}_k(1,m')\mu_{km}, \cdots, \sum_{m\in\mathfrak{G}_k}\boldsymbol{X}_k(N_k,m')\mu_{km}, \mathbf{0}_{N_{k+1}}^{\mathrm{T}}, \cdots, \mathbf{0}_{N_K}^{\mathrm{T}}\right]^{\mathrm{T}} / \sqrt{\sum_{m=1}^{M}\mu_{km}^2},$$

where m' is the index of SNP m in $\boldsymbol{X}_k$. According to the law of large numbers, $\frac{1}{M_k}\sum_{m\in\mathfrak{G}_k}\boldsymbol{X}_k(n',m')\mu_{km}$, where n' is the index of individual *n* in $\boldsymbol{X}_k$ and $n \in \mathfrak{S}_k$, will converge to its mathematical expectation $\frac{1}{M_k}\sum_{m=1}^{M}\mu_{km}^2$ as M_k becomes large.

Therefore, for large M_k

$$\boldsymbol{X}\boldsymbol{v}_k = \sqrt{\sum_{m=1}^{M}\mu_{km}^2}\left[\mathbf{0}_{N_1}^{\mathrm{T}},\cdots,\mathbf{0}_{N_{k-1}}^{\mathrm{T}},\mathbf{1}_{N_k}^{\mathrm{T}},\mathbf{0}_{N_{k+1}}^{\mathrm{T}},\cdots,\mathbf{0}_{N_K}^{\mathrm{T}}\right]^{\mathrm{T}} = s_k\boldsymbol{u}_k,$$

where

$$s_k = \sqrt{N_k\sum_{m=1}^{M}\mu_{km}^2}$$

$$\boldsymbol{u}_k = \left[\mathbf{0}_{N_1}^{\mathrm{T}},\cdots,\mathbf{0}_{N_{k-1}}^{\mathrm{T}},\mathbf{1}_{N_k}^{\mathrm{T}},\mathbf{0}_{N_{k+1}}^{\mathrm{T}},\cdots,\mathbf{0}_{N_K}^{\mathrm{T}}\right]^{\mathrm{T}}/\sqrt{N_k}$$

For the principal score vector $\boldsymbol{a}_k$ that is associated with population *k*,

$$\boldsymbol{a}_k = \boldsymbol{X}\boldsymbol{b}_k = \boldsymbol{X}\boldsymbol{v}_k/\sqrt{\sum_{m=1}^{M}\mu_{km}^2} = \left[\mathbf{0}_{N_1}^{\mathrm{T}},\cdots,\mathbf{0}_{N_{k-1}}^{\mathrm{T}},\mathbf{1}_{N_k}^{\mathrm{T}},\mathbf{0}_{N_{k+1}}^{\mathrm{T}},\cdots,\mathbf{0}_{N_K}^{\mathrm{T}}\right]^{\mathrm{T}}.$$

Proofs of equations (15)-(16) and an approximate maximum likelihood estimate

Consider the M_k SNPs that are specific to population *k*. We have

$$\mathrm{E}\left[\sum_{m\in\mathfrak{G}_k}\boldsymbol{X}(n,m)\right] = \sum_{m\in\mathfrak{G}_k}p_n^k\mu_{km} = p_n^k\sum_{m\in\mathfrak{G}_k}\mu_{km}. \qquad \text{(S-4)}$$

Therefore, the estimate of p_n^k by the method of moment is

$$\hat{p}_n^k = \frac{\sum_{m\in\mathfrak{G}_k}\boldsymbol{X}(n,m)}{\sum_{m\in\mathfrak{G}_k}\mu_{km}}.$$

Log-likelihood function of $p_n^1, p_n^2, \cdots, p_n^K$ is

$$l(p_n^1, p_n^2, \cdots, p_n^K) = \sum_{k=1}^{K}\sum_{m\in\mathfrak{G}_k}\{\boldsymbol{X}(n,m)\ln(2p_n^kf_{km}) + [1-\boldsymbol{X}(n,m)]\ln(1-2p_n^kf_{km})\}.$$

Let

$$\frac{\partial l}{\partial p_n^k} = 0,$$

we have

$$\sum_{m\in\mathfrak{G}_k}\left[\frac{\boldsymbol{X}(n,m)}{p_n^k} - 2f_{km}\frac{1-\boldsymbol{X}(n,m)}{1-2p_n^kf_{km}}\right] = 0,$$

$$p_n^k\sum_{m\in\mathfrak{G}_k}\mu_{km}\frac{1-\boldsymbol{X}(n,m)}{1-p_n^k\mu_{km}} - \sum_{m\in\mathfrak{G}_k}\boldsymbol{X}(n,m) = 0. \qquad \text{(S-5)}$$

Maximum likelihood estimate (MLE) of p_n^k, $k = 1,2 \cdots K$, can be obtained by solving the equation (S-5).

Using the first order approximation $\frac{1}{1-x} \approx 1 + x$, (S-5) becomes

$$p_n^k \sum_{m \in \mathfrak{G}_k} \mu_{km}[1 - \boldsymbol{X}(n,m)][1 + p_n^k \mu_{km}] - \sum_{m \in \mathfrak{G}_k} \boldsymbol{X}(n,m) = 0,$$

which is

$$(p_n^k)^2 \sum_{m \in \mathfrak{G}_k} \mu_{km}^2[1 - \boldsymbol{X}(n,m)] + p_n^k \sum_{m \in \mathfrak{G}_k} \mu_{km}[1 - \boldsymbol{X}(n,m)] - \sum_{m \in \mathfrak{G}_k} \boldsymbol{X}(n,m) = 0.$$

Then, approximate MLE of p_n^k can be obtained by solving the quadratic equation above.

Principal scores are unbiased estimates of the ancestral proportions

According to (12), the principal score of individual *n* associated with population *k* is

$$\boldsymbol{a}_{kn} = \frac{\sum_{m=1}^{M} \mu_{km} \boldsymbol{X}(n,m)}{\sum_{m=1}^{M} \mu_{km}^2} = \frac{\sum_{m \in \mathfrak{G}_k} \mu_{km} \boldsymbol{X}(n,m)}{\sum_{m \in \mathfrak{G}_k} \mu_{km}^2}.$$

Its mathematical expectation can be found as

$$\mathrm{E}[\boldsymbol{a}_{kn}] = \frac{\sum_{m \in \mathfrak{G}_k} \mu_{km} \mathrm{E}[\boldsymbol{X}(n,m)]}{\sum_{m \in \mathfrak{G}_k} \mu_{km}^2} = \frac{\sum_{m \in \mathfrak{G}_k} \mu_{km}^2 p_n^k}{\sum_{m \in \mathfrak{G}_k} \mu_{km}^2} = p_n^k.$$

Therefore, the principal scores $\boldsymbol{a}_k$ are unbiased estimates of the ancestral proportions for population *k*. Note that $\boldsymbol{v}_k$ was chosen to maximize the second-order sample moment of $\boldsymbol{X}\boldsymbol{v}_k$ asymptotically, principal scores tend to have the largest variances amongst linear estimators.

The BLUE of the ancestral proportions

Consider a linear unbiased estimate of p_n^k,

$\hat{p}_n^k = \sum_{m \in \mathfrak{G}_k} \alpha_{km} \boldsymbol{X}(n, m)$,

whose expectation

$\mathrm{E}[\hat{p}_n^k] = \sum_{m \in \mathfrak{G}_k} \alpha_{km} \mathrm{E}[\boldsymbol{X}_k(n, m)]$

$= \sum_{m \in \mathfrak{G}_k} \alpha_{km} p_n^k \mu_{km}$

$= p_n^k \sum_{m \in \mathfrak{G}_k} \alpha_{km} \mu_{km}$

$= p_n^k$.

Therefore, we have

$\sum_{m \in \mathfrak{G}_k} \alpha_{km} \mu_{km} = 1.$

Its variance

$\mathrm{Var}[\hat{p}_n^k] = \sum_{m \in \mathfrak{G}_k} \alpha_{km}^2 \mathrm{Var}[\boldsymbol{X}_k(n, m')]$

$= \sum_{m \in \mathfrak{G}_k} \alpha_{km}^2 p_n^k \mu_{km} (1 - p_n^k \mu_{km})$

$\approx p_n^k \sum_{m \in \mathfrak{G}_k} \alpha_{km}^2 \mu_{km}$,

where the approximation holds for small MAFs. To find the BLUE of p_n^k, we solve the following optimization problem.

$\min_{\alpha_{km}, m \in \mathfrak{G}_k} \sum_{m \in \mathfrak{G}_k} \alpha_{km}^2 \mu_{km}$ subject to $\sum_{m \in \mathfrak{G}_k} \alpha_{km} \mu_{km} = 1$.

Using the Lagrangian method, the solution is given by solving the following linear equations

$$\begin{pmatrix} 2\boldsymbol{A}_k & \boldsymbol{\mu}_k \\ \boldsymbol{\mu}_k^{\mathrm{T}} & 0 \end{pmatrix} \begin{pmatrix} \boldsymbol{\alpha}_k^* \\ \lambda^* \end{pmatrix} = \begin{pmatrix} \boldsymbol{0}_{M_k} \\ 1 \end{pmatrix},$$

where

$\boldsymbol{\mu}_k = (\cdots, \mu_{km}, \cdots)^{\mathrm{T}},\ m \in \mathfrak{G}_k$,

$\boldsymbol{A}_k = \mathrm{diag}(\boldsymbol{\mu}_k)$,

and λ is the Lagrangian multiplier. The optimal loading vector $\boldsymbol{\alpha}_k^*$ that yields the BLUE of p_n^k is

$$\boldsymbol{\alpha}_k^* = \boldsymbol{A}_k^{-1}\boldsymbol{\mu}_k / \boldsymbol{\mu}_k^{\mathrm{T}}\boldsymbol{A}_k^{-1}\boldsymbol{\mu}_k$$

$$= \mathbf{1}_{M_k} / \sum_{m\in\mathfrak{G}_k} \mu_{km} \ .$$

Compared with (17)-(19), this is the estimate of p_n^k by the method of moment.

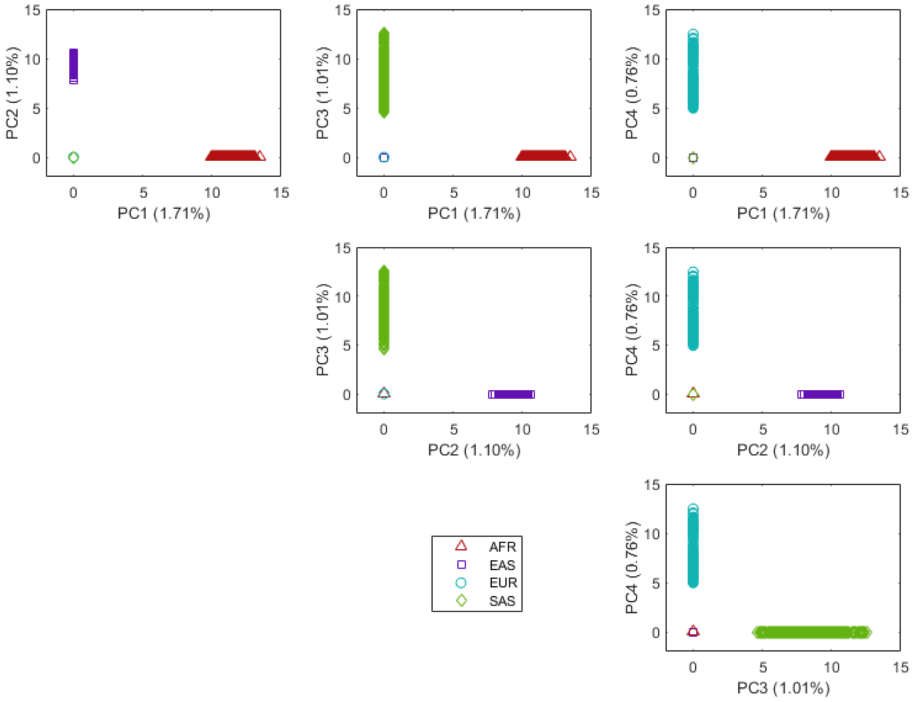

Fig. S1. PC 1-PC 4 of the four reference populations in the 1kGP using 4 reference populations.

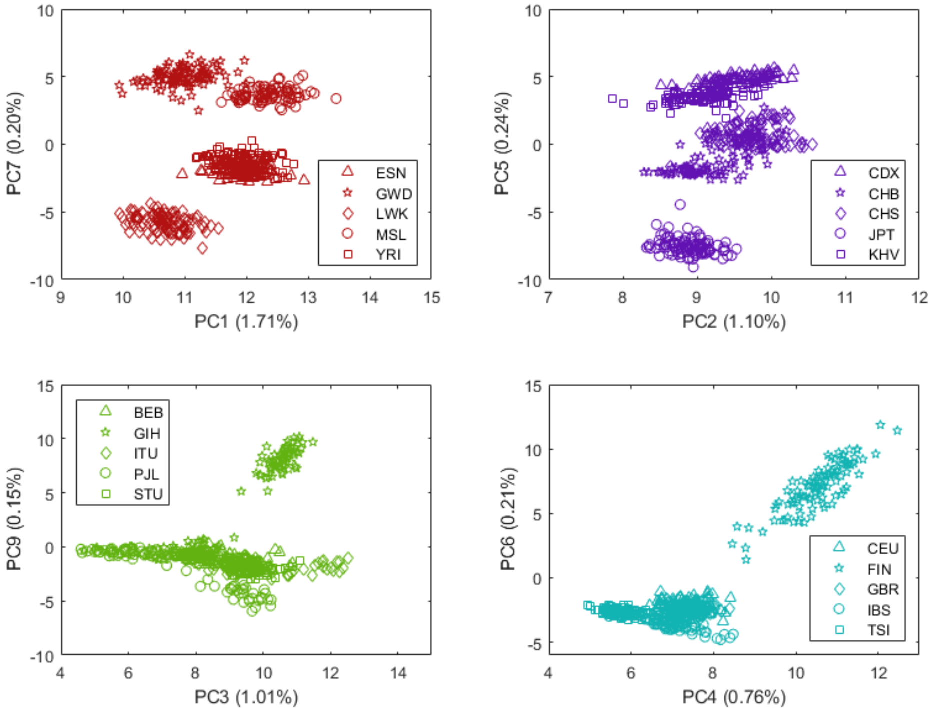

Fig. S2. PC 5, 6, 7 and 9 of the four reference populations in the 1kGP using 4 reference populations.

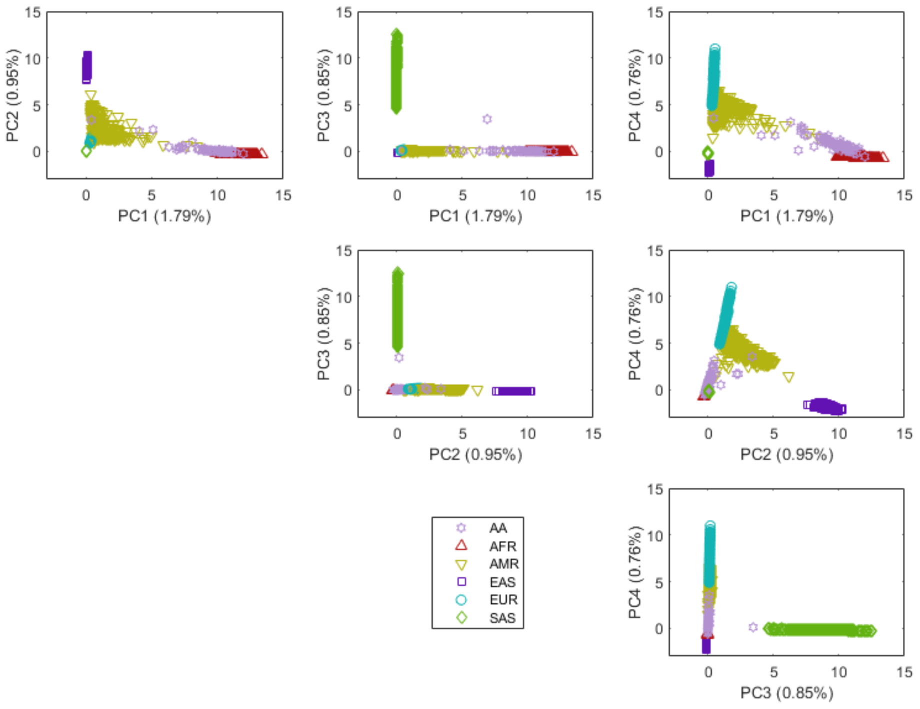

Fig. S3. PC 1-PC 4 of 2504 individuals in the 1kGP using 4 reference populations.

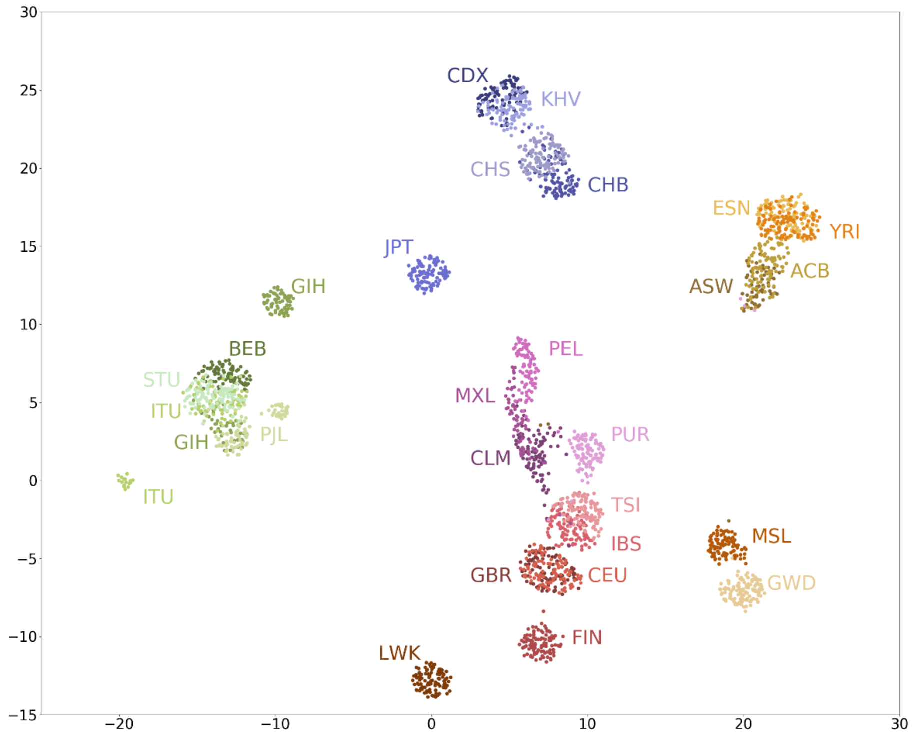

Fig. S4. UMAP analysis of the top 20 PCs in the 1kGP data with common SNPs.

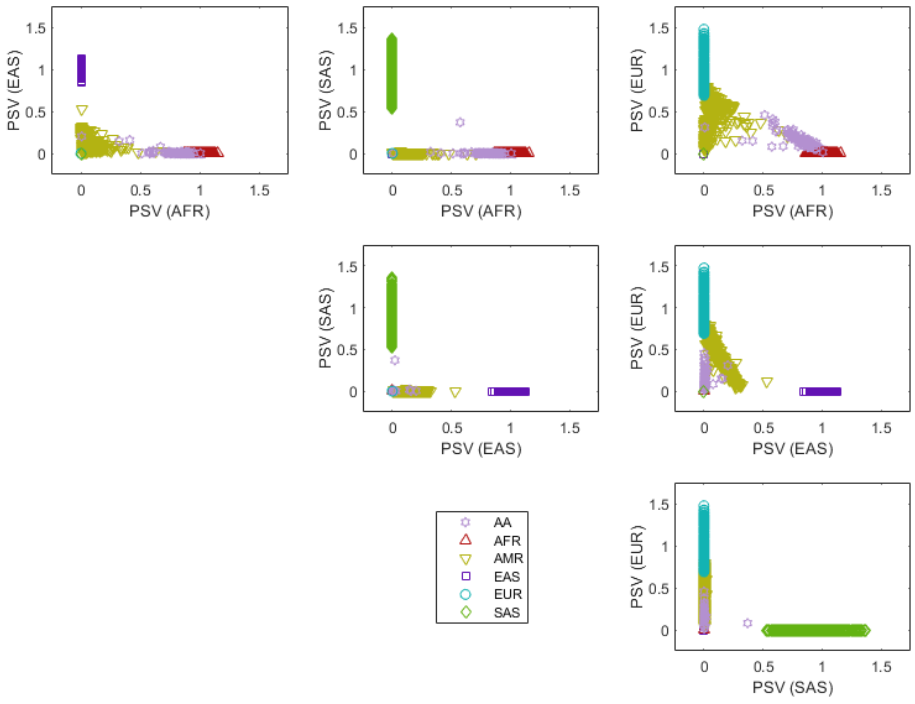

Fig. S5. Principal scores of 2504 individuals in the 1kGP using 4 reference populations. PSV: Principal score vector.

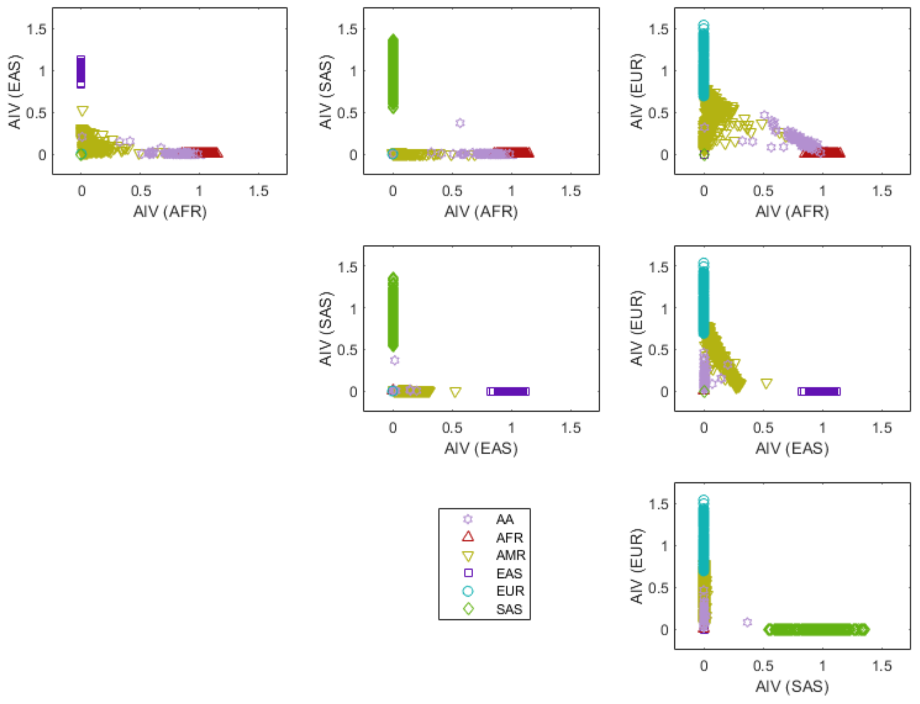

Fig. S6. Ancestral information of 2504 individuals in the 1kGP using 4 reference populations. AIV: Ancestral information vector.

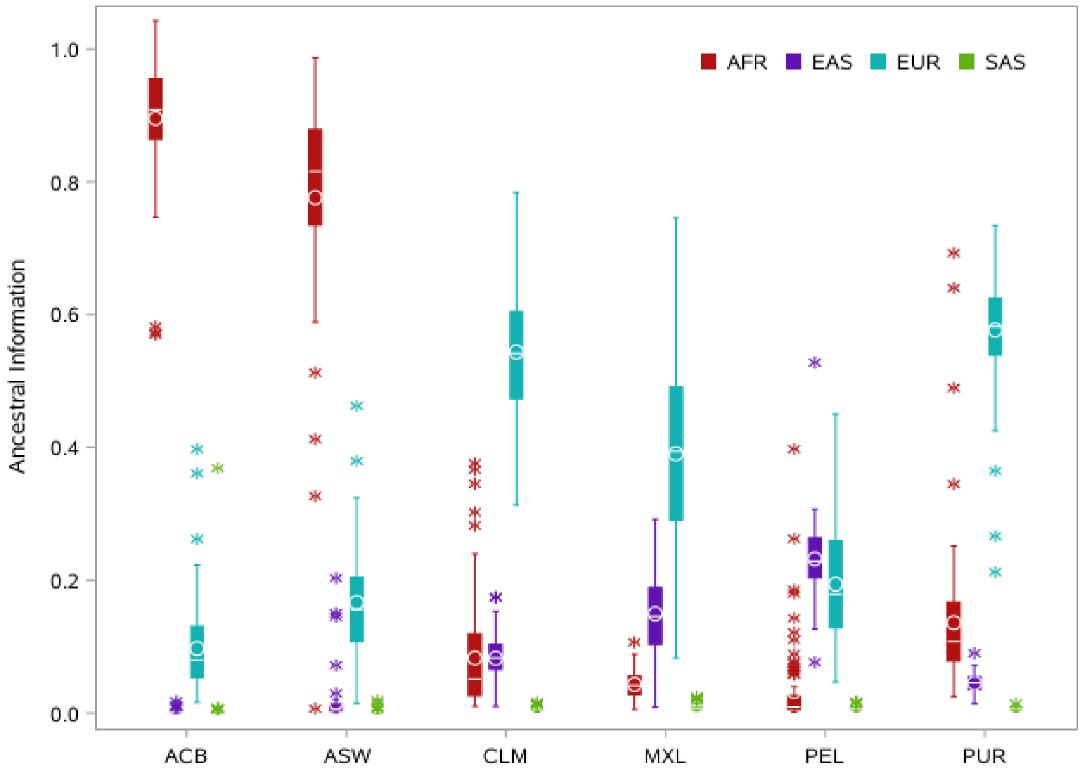

Fig. S7. Box plot of ancestral information of admixed populations in the 1kGP using 4 reference populations.

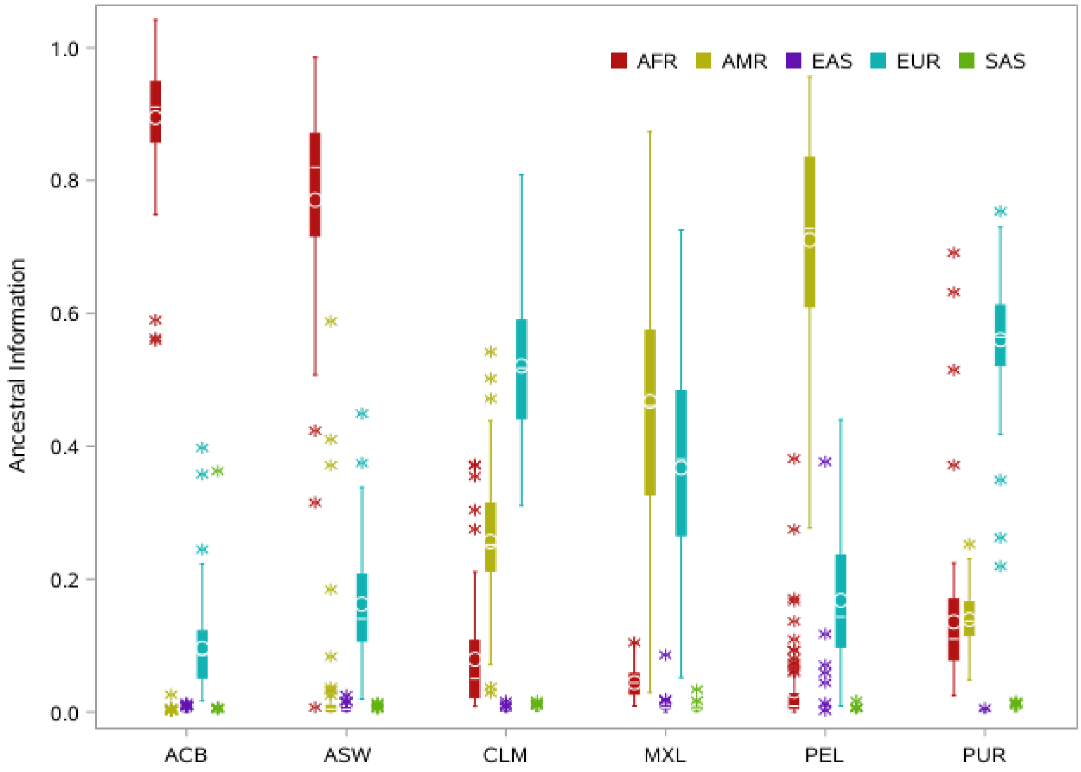

Fig. S8. Box plot of ancestral information of admixed populations in the 1kGP using 5 reference populations.

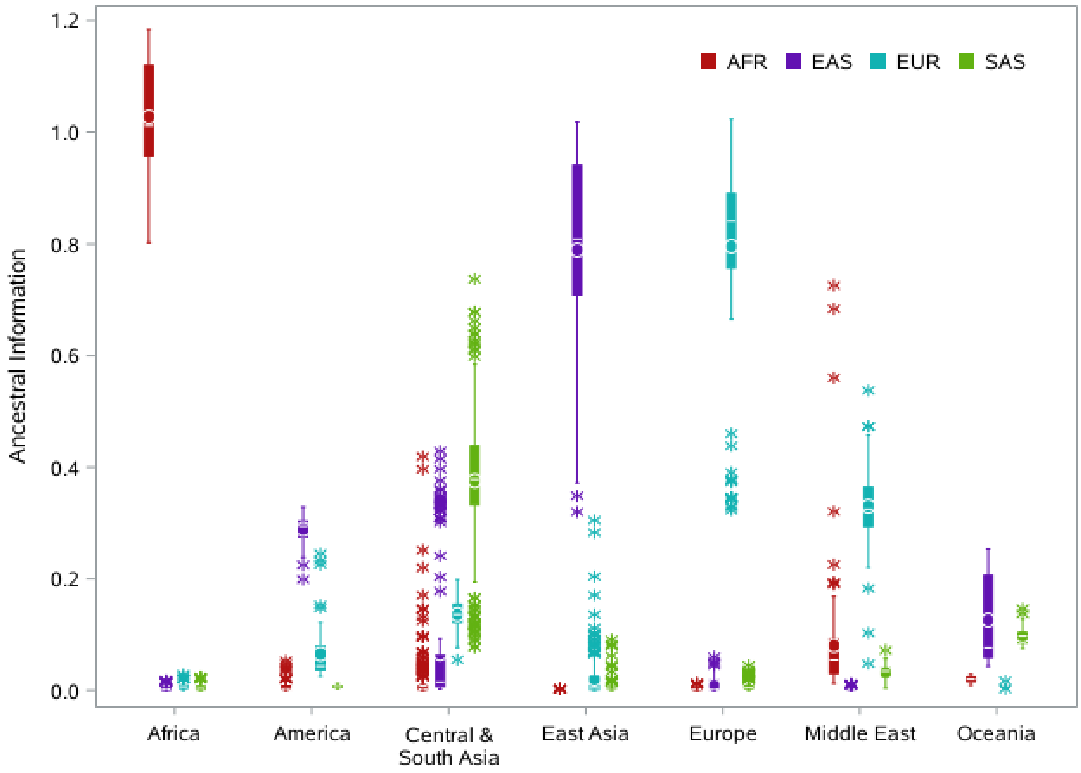

Fig. S9. Box plot of ancestral information of 929 individuals in the HGDP using 4 reference populations.

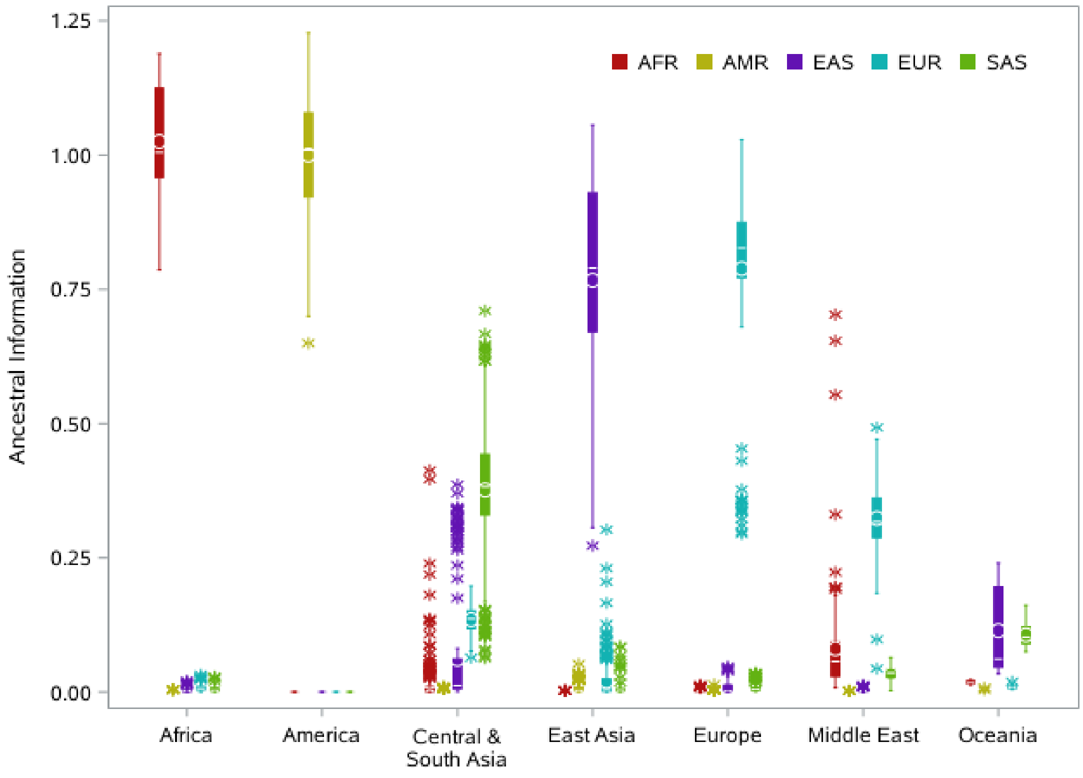

Fig. S10. Box plot of ancestral information of 929 individuals in the HGDP using 5 reference populations.

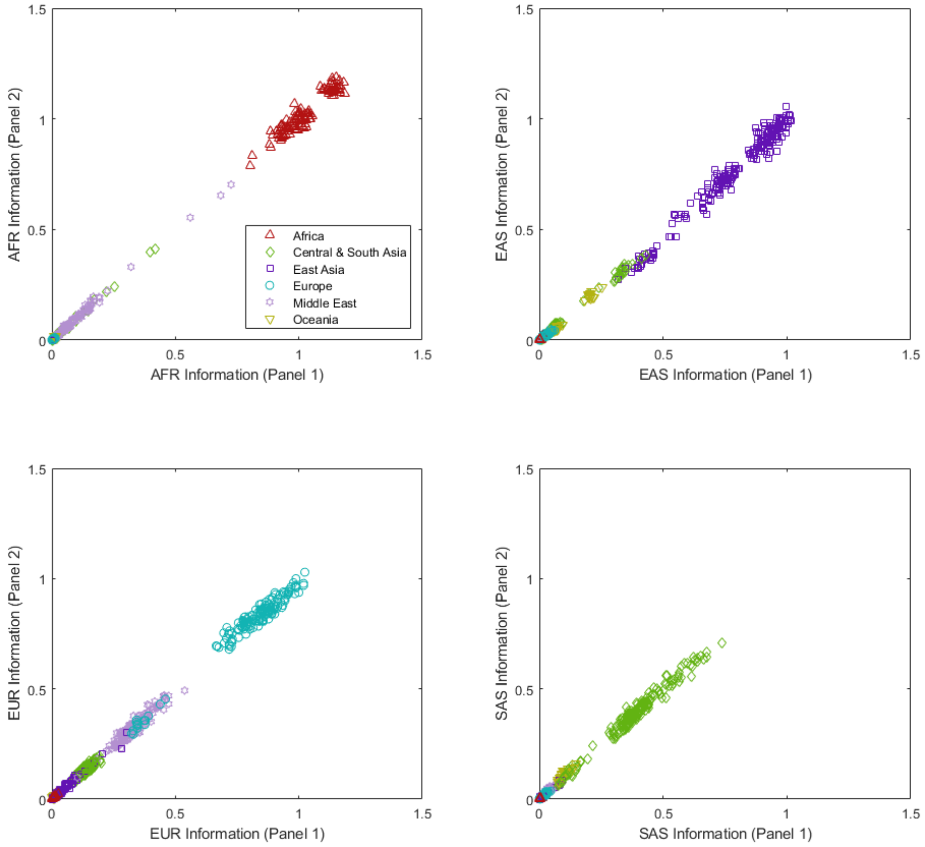

Fig. S11. AFR, EAS, EUR and SAS information with two panels of population-specific SNPs in 868 non-AMR individuals from the HGDP.

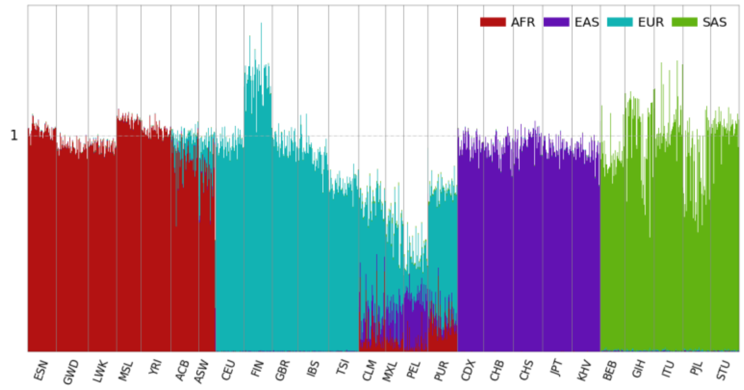

Fig. S12. Ancestral spectra of 2504 individuals in the 1kGP with population-specific SNPs from phase 3 data.

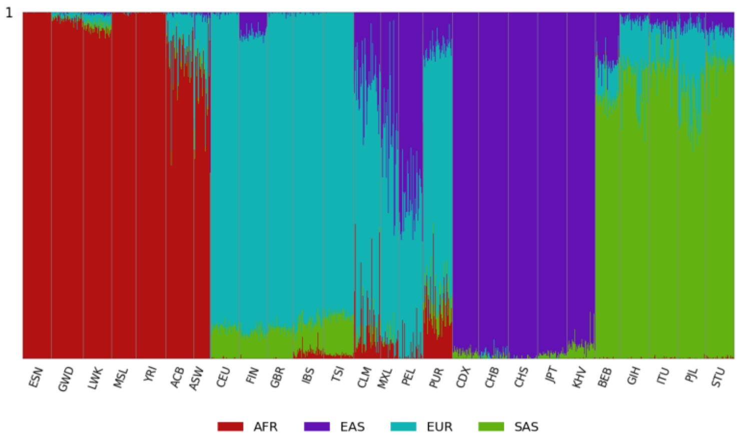

Fig. S13. Unsupervised ADMIXTURE analysis (*K*=4) of 2504 individuals in the 1kGP with common SNPs.

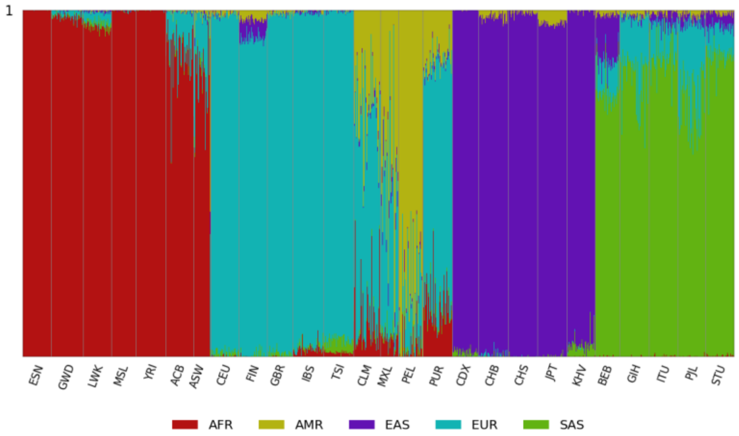

Fig. S14. Unsupervised ADMIXTURE analysis (*K*=5) of 2504 individuals in the 1kGP with common SNPs.

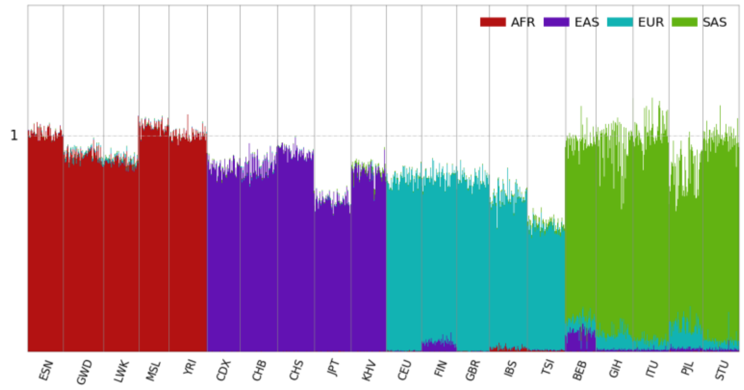

Fig. S15. Ancestral spectra of AFR, EAS, EUR and SAS populations in the 1kGP using 4 reference populations.

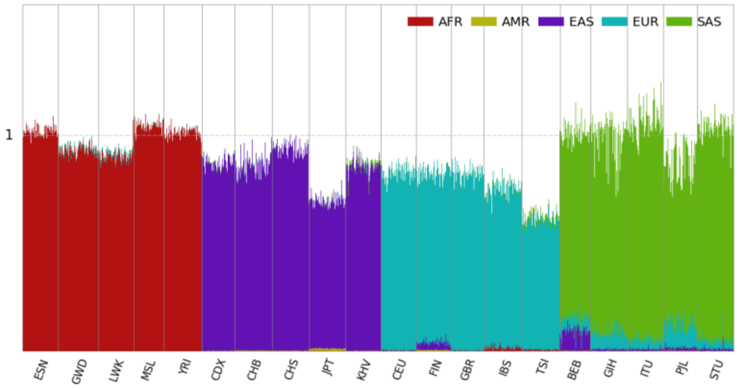

Fig. S16. Ancestral spectra of AFR, EAS, EUR and SAS populations in the 1kGP using 5 reference populations.

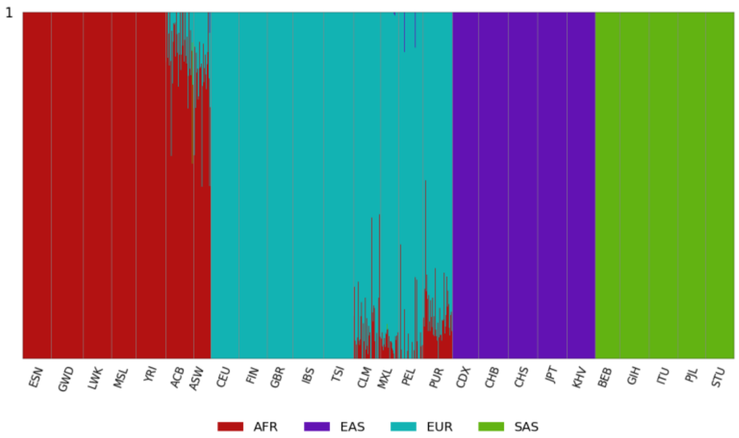

Fig. S17. Supervised ADMIXTURE analysis using 4 reference populations.

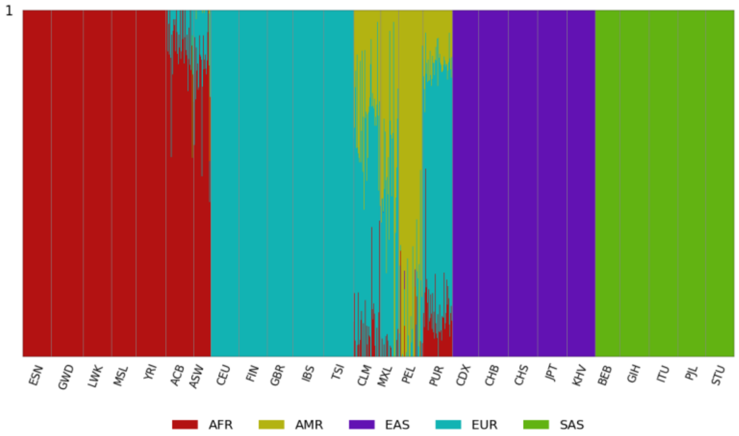

Fig. S18. Supervised ADMIXTURE analysis using 5 reference populations.
